# Simulating Molecular Single Vibronic Level Fluorescence Spectra with Ab Initio Hagedorn Wavepacket Dynamics

**DOI:** 10.1021/acs.jctc.5c01097

**Published:** 2025-09-15

**Authors:** Zhan Tong Zhang, Jiří J. L. Vaníček

**Affiliations:** Laboratory of Theoretical Physical Chemistry, Institut des Sciences et Ingénierie Chimiques, 27218 Ecole Polytechnique Fédérale de Lausanne (EPFL), CH-1015 Lausanne, Switzerland

## Abstract

We present a practical, ab initio time-dependent method using Hagedorn wavepackets to efficiently simulate single vibronic level (SVL) fluorescence spectra of polyatomic molecules from arbitrary initial vibrational levels. We apply the method to compute SVL spectra of anthracene by performing wavepacket dynamics on a 66-dimensional harmonic potential energy surface constructed from density functional theory calculations. The Hagedorn approach captures both mode distortion (frequency changes) and mode mixing (Duschinsky rotation) within the harmonic approximation. We not only reproduce the previously reported simulation results for singly excited 12^1^ and 1̅1̅^1^ levels but are also able to compute SVL spectra from multiply excited levels in good agreement with experiments. Notably, all spectra were obtained from the same wavepacket trajectory without any additional propagation beyond what is required for the emission spectrum from the ground vibrational level of the electronically excited state.

## Graphical abstract

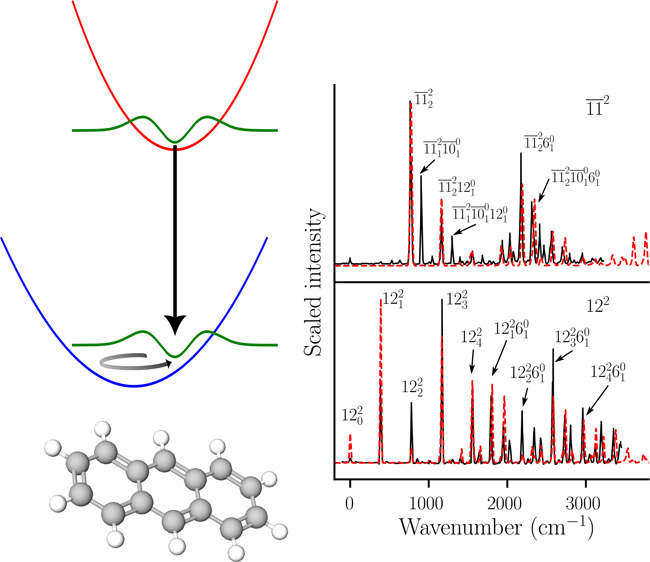

## 1 Introduction

Single vibronic level (SVL) spectroscopy measures the fluorescence decay of a system following a vibronic excitation to a specific vibrational level in the excited electronic state. This spectroscopic tool has played an important role in investigations of excited-state relaxation pathways,^1–4^ vibrational structures of electronic states,^3,5,6^ and in the identification and characterization of rotamers and reactive intermediates.^7–9^ Computational methods based on ab initio electronic structure calculations have been developed and implemented to better understand these spectra and guide experiments.^10–16^

Calculations of SVL spectra have mostly relied on the time-independent approach, where individual Franck–Condon factors are computed for each vibronic transition.^10,11,16,17^ However, for larger polyatomic molecules, the task becomes challenging due to the large number of possible transitions and the computation of nonseparable overlap integrals.^18–21^ The time-dependent approach, which is more natural for low- or intermediate-resolution spectra, avoids the need to prescreen transitions and can more efficiently accommodate Duschinsky rotation (mode mixing),^14,22,23^ anharmonicity,^24–27^ Herzberg–Teller,^22,25,26,28–33^ and temperature effects^34–38^ in both linear and multidimensional^39–42^ vibronic spectra.

In the first practical implementation of the time-dependent approach to SVL spectra,^28^ Tapavicza developed a generating-function-based method to simulate emissions from a singly excited state (i.e., with at most a single vibrational excitation in one mode only).^14^ Using a displaced, distorted, and Duschinsky-rotated global harmonic model, he successfully computed SVL spectra of singly excited anthracene in good agreement with the experimental results.^3^

In order to evaluate SVL spectra associated with emission from arbitrary vibronic levels, we recently proposed^43^ another time-dependent approach, one based on Hagedorn functions.^44–46^ These functions in the form of a Gaussian multiplied by carefully constructed polynomials^46–48^ are exact solutions of the time-dependent Schrödinger equation with a quadratic potential and have drawn attention in applied mathematics^45–47,49–55^ because of their promising applications in physics and chemistry.^56–60^

The recursive expressions we derived for their overlap integrals^61^ made it possible to apply Hagedorn wavepackets in computational spectroscopy.^43,62^ While Hagedorn functions, as a complete orthonormal basis, can represent initial states of arbitrary shape, their form is particularly well-suited for simulating SVL spectra. Within the harmonic and Condon approximations, the SVL process may be represented by a single Hagedorn function at all times and provides a clear, uncomplicated first demonstration of the Hagedorn dynamics approach to vibronic spectroscopy. In ref 43, we validated the Hagedorn approach to compute SVL spectra against quantum split-operator results in two-dimensional harmonic model systems incorporating displacement, mode distortion (changes of vibrational frequencies in different electronic states), and mode mixing (Duschinsky rotation) effects.

Here, we describe how the approach from ref 43 extends to realistic polyatomic molecules within the global harmonic approximation, which provides a convenient starting point for studying larger molecular systems and can guide the selection of ab initio methods for further on-the-fly local harmonic studies that include anharmonic effects.^62^ In order to implement ab initio Hagedorn wavepacket dynamics, we neglect ro-vibrational coupling and perform the vibrational dynamics in the normal-mode coordinates on a full-dimensional, global harmonic potential energy surface derived from electronic structure calculations. As a test, we simulate the SVL spectra of anthracene from 1̅1̅^*j*^ and 12^*j*^ (*j* = 1, 2) levels, for which assigned experimental data are available.^3^ We also predict the SVL spectra from the 12^1^6^1^, 12^1^5̅^1^, and 12^1^5^1^ levels, which remain to be measured experimentally. Whereas the singly excited cases were already successfully simulated by Tapavizca,^14^ our Hagedorn approach can treat higher and mixed excitations from a single semiclassical trajectory by computing overlaps between Hagedorn functions at a small additional cost that becomes negligible in on-the-fly local harmonic applications.

## 2 Methodology

### 2.1 Time-Dependent Approach to Spectroscopy

The traditional “sum-over-states” approach for evaluating vibronic spectra relies on computing the Franck–Condon overlaps between the initial and final vibrational states. In displaced and distorted harmonic potentials, these overlap integrals can be evaluated exactly using analytical formulas,^63,64^ or, when Duschinsky effects are considered, through recursive procedures.^65–67^ While this time-independent approach provides an explicit contribution from each vibronic transition to the spectrum, computational cost becomes prohibitive in larger molecules if one does not restrict the number of final vibrational states included in the calculation (either heuristically or through an automated procedure implemented in packages such as FCclasses^66,67^ ).

An alternative, time-dependent approach avoids the need to preselect and compute individual transitions. The spectrum is instead evaluated as the Fourier transform of an appropriate wavepacket autocorrelation function

$$C(t)=\langle \psi_{0}|\psi_{t}\rangle$$ 1

i.e., the overlap between the initial nuclear wavepacket ψ_0_ and the wavepacket ψ_*t*_ propagated to time *t* on the final electronic surface. In the case of SVL fluorescence spectrum, the emission rate per unit frequency from a vibrational level |ψ_0_⟩ = |*K*⟩ of the excited electronic state *e* is given by

σem(ω)=4ω33πℏc3|μge|2Re∫0∞C(t)®exp[it(ω−ωe,K)]dt 2

where *K* ≡ (*K*_1_, ···, *K*_*D*_) is a multi-index specifying the initial vibrational quantum numbers in the *D* normal modes, μ_*ge*_ is the electronic transition dipole moment (a constant scalar within the Condon approximation), and ℏω_*e*,*K*_ is the energy of the initial vibronic state. The nuclear wavepacket |ψ_*t*_⟩ = exp(−i*Ĥ*_*g*_ *t*/*ℏ*)|ψ_0_⟩ in eq 1 is propagated with the ground-state Hamiltonian *Ĥ*_*g*_.

In contrast to the time-independent approach, the dynamics-based approach does not explicitly enumerate the final vibrational states in the vibronic spectrum and obtains all peaks at once. The convergence of the spectrum (in terms of the frequency range and resolution) is determined by the time step used for evaluating autocorrelation functions and the total time of propagation. Although the individual contributions of each transition are not directly available, the time-dependent approach is more natural and computationally straightforward when one is interested in low- to intermediate-resolution spectra. It also enables the treatment of more general initial states (e.g., thermal ensembles or non-Condon effects) and potential energy surfaces (e.g, Duschinsky rotation and anharmonicity).

### 2.2 Representation of SVL Initial States by Hagedorn Functions

For the conventional emission spectrum from the ground level *K* = **0**, the initial vibrational state may be represented by a normalized *D*-dimensional Gaussian wavepacket

$$\varphi_{0}(q)=\frac{1}{(\pi \hbar {)}^{D/4}\sqrt{\det(Q_{t})}}\;\exp\left[\frac{i}{\hbar }\left(\frac{1}{2}x^{T}\cdot P_{t}\cdot Q_{t}^{-1}\cdot x+p_{t}^{T}\cdot x+S_{t}\right)\right]$$ 3

in Hagedorn’s parametrization, where *x* ≔ *q* – *q*_*t*_ is the shifted position, *q*_*t*_ and *p*_*t*_ are the position and momentum of the center of the wavepacket, *S*_*t*_ is the classical action, and *Q*_*t*_ and *P*_*t*_ are complex-valued *D*-dimensional matrices that satisfy the so-called symplecticity conditions^46,53^ (see eqs 5 and 6 of ref 43) and determine the width matrix A_*t*_ ≔ *P*_*t*_·*Q*_*t*_^–1^ of the Gaussian. We can then apply Hagedorn’s raising operator

$$A^{†}≔\frac{i}{\sqrt{2\hbar }}(P_{t}^{†}\cdot (\hat{q}-q_{t})-Q_{t}^{†}\cdot (\hat{p}-p_{t}))$$ 4

to the Gaussian wavepacket φ_0_ to recursively generate an orthonormal family of Hagedorn functions

$$\varphi_{K+\langle j\rangle }=\frac{1}{\sqrt{K_{j}+1}}A_{j}^{†}\varphi_{K}$$ 5

in the form of multivariate polynomials multiplied by a common Gaussian, in which

$$\left\langle j\rangle =(\underset{j-1}{\underset{︸}{0,\cdot \cdot \cdot ,0}},1,\underset{D-j}{\underset{︸}{0,\cdot \cdot \cdot ,0}}\right)$$

is the *D*-dimensional unit vector along the *j*th degree of freedom.^44,46,53,68^

The initial vibrational state |ψ_0_⟩ = |*K*⟩ in the SVL emission process can be exactly represented by a single Hagedorn function φ_*K*_ in the normal-mode coordinates. In the harmonic approximation, normal-mode coordinates for a given electronic state diagonalize the Hessian at its equilibrium geometry, and the ground vibrational wave function is a Gaussian (eq 3) with a diagonal width matrix A_0_ = *P*_0_·*Q*_0_^–1^. With both *Q*_0_ and *P*_0_ diagonal, the associated multidimensional Hagedorn function is a product of univariate functions, each of which is a Gaussian multiplied by a Hermite polynomial. As we will see in the next sections, this simple Hermite factorization is lost during the dynamics on a general harmonic surface.

### 2.3 Hagedorn Wavepacket Dynamics

Remarkably, Hagedorn wavepackets are exact solutions to the time-dependent Schrödinger equation (TDSE) with a harmonic potential

$$V_{g}(q)=v_{0,g}+(q-q_{\mathrm{ref},g}{)}^{T}\cdot \kappa_{g}\cdot (q-q_{\mathrm{ref},g})/2$$ 6

which, in the calculations of emission spectra, represents the potential energy surface of the ground electronic state. The reference position *q*_ref,*g*_, the reference energy *v*_0,*g*_, and the Hessian matrix κ_*g*_ may be determined from ab initio electronic structure calculations. The time evolution of a Hagedorn wavepacket then follows particularly simple, classical-like equations:

$$\begin{array}{l} {\mathrm{q̇}}_{t}=m^{-1}\cdot p_{t},,{ṗ}_{t}=-V_{g}^{\prime }(q_{t})=-\kappa_{g}\cdot (q_{t}-q_{\mathrm{ref}}),\\ {\mathrm{Q̇}}_{t}=m^{-1}\cdot P_{t},,{Ṗ}_{t}=-V_{g}^{\prime \prime }(q_{t})\cdot Q_{t}=-\kappa_{g}\cdot Q_{t},\\ {Ṡ}_{t}=L_{t}=p^{T}\cdot m^{-1}\cdot p/2-V_{g}(q_{t}) \end{array}$$ 7

where *m* is the mass matrix and *L*_*t*_ is the Lagrangian.^46,53,69^ The multi-index *K* of the Hagedorn function does not change during the exact propagation in up-to-quadratic potentials, i.e., the Hagedorn function propagated in a harmonic potential to time *t* retains its form,

$$\varphi_{K,t}=(K!{)}^{-1/2}(A^{†}[q_{t},p_{t},Q_{t},P_{t}]{)}^{K}\varphi_{0}[q_{t},p_{t},Q_{t},P_{t},S_{t}]$$ 8

even in the presence of mode mixing (when Hessian κ_*g*_ is not a diagonal matrix). In fact, the equations of motion given by eqs 7 are the same as in the thawed Gaussian approximation,^70,71^ which has been applied to vibronic spectra from ground vibrational levels^23,24,72,73^ and extended to propagate not only Gaussians but also Gaussians multiplied by a linear polynomial prefactor.^30,32,36,74–76^ Using Hagedorn wavepackets, it is possible to treat arbitrary polynomials times a Gaussian (describing, e.g., excited vibrational states), and since the propagation (eqs 7) still depends only on the Gaussian’s parameters, a single Gaussian trajectory is sufficient to obtain SVL spectra from all initial vibrational levels.

Although eqs 7 can also be solved analytically to obtain a solution at an arbitrarily long time *t* in a harmonic system,^77^ the spectrum (eq 2) depends on the values of the autocorrelation function *C*(*t*) at all times *t* from 0 to *t*_max_ (the time that gives the desired spectral resolution). Thus, compared to evaluating analytical solutions at each discretized time point over the total time of propagation, numerical propagation (with geometric integrators) is nearly as accurate and efficient. In high-dimensional harmonic systems, the computational cost of evaluating SVL spectra is dominated by computing the Hagedorn overlaps rather than the propagation, which only needs to be performed once for all initial states.

The numerical approach to wavepacket propagation becomes necessary when the Hagedorn dynamics is combined with the local harmonic approximation to take into account anharmonicity in flexible molecules. There, as in the thawed Gaussian approximation, only minimal modifications need to be made to eqs 7 by evaluating the gradient *V*_*g*_^′^(*q*_*t*_) and Hessian *V*_*g*_^″^(*q*_*t*_) on the fly from electronic structure calculations (instead of from the static Hessian matrix κ_*g*_ in the global harmonic model), but analytical solutions at arbitrary times are no longer possible. In the local harmonic case, the cost of evaluating SVL spectra is dominated by ab initio calculations and the cost of computing overlaps (in postprocessing) becomes negligible.

Whereas closed-form analytical expressions exist for overlaps between two Gaussian wavepackets, computing the SVL spectra requires the overlaps between two “excited” Hagedorn functions associated with two different Gaussian centers, a problem similar to the computation of Franck–Condon factors in harmonic systems in the time-independent approach. In ref 61, we derived recurrence relations for algebraically evaluating the overlaps between any Hagedorn functions; these expressions are also presented succinctly in the Supporting Information here. Although more complex than simple Gaussian overlaps, these calculations typically involve only one or two initially excited modes containing just a few vibrational quanta.

Beyond the *O*(*D* ^3^) scaling with the number of normal modes *D* (the same as in computing overlaps of Gaussian), the cost of evaluating the overlaps between Hagedorn functions depends on the number of excited modes and the vibrational excitation levels. For multiply excited levels in a single mode, the cost of evaluating ⟨φ_*K*,*t*=0_|φ_*K*,*t*_⟩ scales quadratically with the excitation *K*.

Compared to the sum-over-states approach, which requires overlaps between an initial vibrational excited state and a considerable number of possible final states in high-dimensional systems, the time-dependent Hagedorn method requires only overlaps between Hagedorn functions with the same multi-index *K* (determined by the initial vibrational excitation). The recursive nature of the overlap expressions also means that computing autocorrelation functions for multiply excited levels inherently generates those for lower levels encountered during recursion (e.g., computing the autocorrelation for a doubly excited level also provides results for singly excited levels). Conversely, with efficient caching implemented, results for lower excitation levels can be reused for higher excitation levels.

### 2.4 Combination with Ab Initio Electronic Structure Calculations

The vibrational nuclear dynamics represented by Hagedorn wavepackets is described in terms of vibrational normal-mode coordinates, which naturally emerge from the harmonic approximation of the potential. In order to make the Hagedorn approach practical for ab initio applications in real molecules, the initial state φ_*K*_ and the potential energy surface (eq 6) must be constructed from electronic structure calculations. Because these calculations are typically carried out in Cartesian or internal coordinates, the computed molecular geometries and Hessians must be first transformed to normal-mode coordinates. For simplicity of propagation, we adopt mass-weighted coordinates so that each normal mode has an identical mass and the mass matrix *m* becomes scalar.

The first step in transforming Cartesian coordinates to mass-weighted normal-mode coordinates is minimizing the ro-vibrational coupling relative to a reference geometry. This is achieved by moving to the center-of-mass frame and applying the Kabsch algorithm to satisfy the translational and rotational Eckart conditions.^78–80^ Next, the normal modes and the corresponding transformation matrix are found by diagonalizing the mass-weighted Hessian matrix of the reference state and projecting out the rotational and translational degrees of freedom (see Section 6.7 of ref 81). In the SVL application, the reference geometry and Hessian are taken from the initial, excited electronic state so that each vibrational eigenstate |*K*⟩ may be represented by a single Hagedorn function φ_*K*_. The diagonalized vibrational Hessian κ_*e*_ in the excited-state normal-mode coordinates determines the width matrix $A_{0}=i\sqrt{m^{1/2}\cdot \kappa_{e}\cdot m^{1/2}}$ of the Gaussian φ_0_ associated with the SVL initial states. In Hagedorn’s parametrization, we set *Q*_0_ = (Im A_0_)^−1/2^ and *P*_0_ = A_0_·*Q*_0_ to satisfy the symplecticity relations.

In the adiabatic harmonic approximation, the Hessian κ_*g*_ defining the ground-state potential (eq 6) used for propagation is evaluated at the ground-state equilibrium geometry *q*_ref,*g*_ = *q*_eq,*g*_; both the geometry and Hessian are transformed to be in the excited-state mass-weighted normal-mode coordinates. In the vertical harmonic approximation, the ground-state Hessian κ_*g*_ is instead evaluated at the optimized excited-state geometry *q*_ref,*g*_ = *q*_eq,*e*_. Due to mode mixing, κ_*g*_ is in general not diagonal when expressed in the excited-state normal-mode coordinates. Therefore, the initially diagonal *Q*_*t*=0_ matrix of the SVL initial state will no longer be diagonal after evolution with the ground-state potential, and the propagated Hagedorn function will not remain a simple product of univariate Hermite functions.^46–48^ However, a single Hagedorn function still suffices to represent the SVL process exactly in the harmonic limit. In contrast, basis expansion methods (using, e.g., products of Gaussians and Hermite polynomials) typically require many functions to maintain accuracy in the presence of mode mixing in multidimensional systems.

## 3 Computational Details

To compare with previously reported results and to validate our method, we used the same density functional theory (DFT) method (at PBE0/def2-TZVP level of theory^82,83^ ) as in ref 14 to construct the global harmonic models. Geometry optimizations and frequency calculations were performed using the Gaussian 16 package^84^ for the ground (*S*_0_^1^A_g_) and the first excited (*S*_1_^1^B_2u_) electronic states of anthracene. The excited-state calculations were carried out using standard linear-response time-dependent DFT. While this level of theory is somewhat simple and approximate, it was shown^14^ to reproduce emission spectra from the ground and singly excited levels reasonably well with results similar to those obtained from more expensive second-order approximate coupled-cluster calculations. The optimized structures of the two states and the frequencies of the vibrational modes analyzed in this work are listed in the Supporting Information. The assignment of the vibrational modes and their symmetry follows the convention of ref 3 and the Supporting Information of ref 14.

A second-order TVT geometric integrator^53,69,85^ was used to propagate the parameters of the Gaussian for a total time of 8 × 10^5^ au (∼19 ps) with a time step of 8 au in the global harmonic systems considered in this work. After the (single!) trajectory of the parameters of the Gaussian was obtained in a given harmonic system, the autocorrelation functions between Hagedorn wavepackets were computed every four steps for each initial vibrational level (0^0^, 12^1^, 12^2^, 1̅1̅^1^, 1̅1̅^2^, 12^1^6^1^, 12^1^5̅^1^, and 12^1^5^1^) using the algebraic algorithm described in ref 61 (a Python implementation is provided in the Supporting Information of ref 43). Given the limited range of the observed experimental data, it was unnecessary to extend the range of the simulated spectra by computing the autocorrelation function at every step. A Gaussian damping function with a half-width at half-maximum of 20,000 au was applied to the autocorrelation functions before performing the Fourier transform.

To facilitate the comparison, the intensities of the highest peaks in all spectra (simulated and experimental) were set to unity. A horizontal shift, determined by aligning the 0_0_^0^ peak in the computed ground-level (0^0^) fluorescence spectrum (see the Supporting Information) to be at the experimental origin 27709 cm^–1^, was applied to the wavenumbers of the simulated spectra in global harmonic potentials to correct for the relatively large error (compared to the vibrational features) in the computed electronic excitation energy; the ω^3^ factor in eq 2 was then adjusted based on the corrected wavenumbers (see the Supporting Information of ref 62 for details).

The details of the artificially modified harmonic systems (analyzed in Figure 3) and of the local harmonic calculations (shown in Figure 4) are available in the Supporting Information.

## 4 Results and Discussion

Anthracene belongs to the *D*_2*h*_ point group. Within the Condon approximation, only vibrational levels belonging to the a_g_ representation can be prepared in the excited ^1^B_2u_ state from the ground vibrational level of the ground ^1^A_g_ state to serve as the initial states in SVL experiments.^3,14^ However, through Herzberg–Teller intensity borrowing, the preparation of SVL initial states with b_1g_ vibrational symmetry is also possible. Out of the 66 vibrational modes, 12 have a_g_ symmetry and 11 have b_1g_ symmetry (denoted by a bar in the mode label). As examples, we show below the SVL spectra with initial vibrational excitations in the a_g_ mode 12 and b_1g_ mode 1̅1̅.

Figure 1 compares the spectra of anthracene evaluated with the Hagedorn approach in the adiabatic harmonic model to the experimental emission spectra from the 12^1^, 12^2^, 1̅1̅^1^, and 1̅1̅^2^ levels.^3^ For these SVL spectra, the Franck–Condon selection rules for emissions provide that transitions from a_g_ levels (12^1^, 12^2^, and 1̅1̅^2^) are only allowed to a_g_ levels, and that transitions from the b_1g_ level (1̅1̅^1^) are only allowed to b_1g_ levels. The total quantum numbers in b_1g_ modes must thus retain the same parity (odd or even) after the transition.

**Figure 1:**
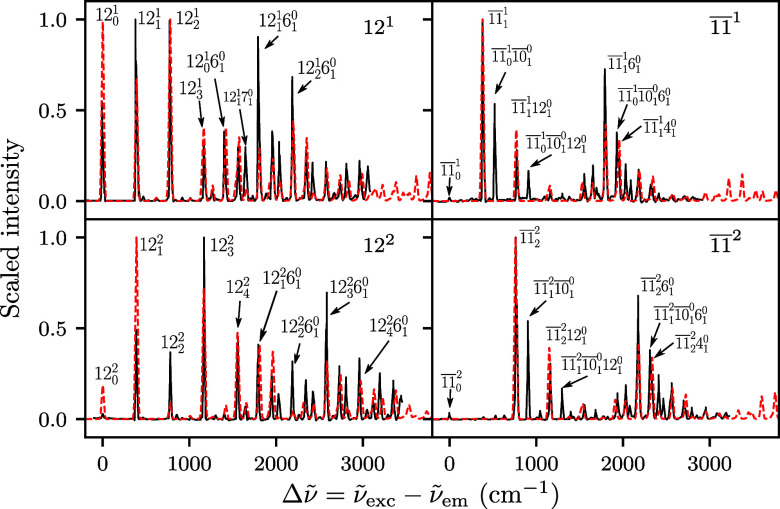
SVL fluorescence spectra of anthracene from initial vibrational levels 12^*j*^ and 1̅1̅^*j*^ (*j* = 1, 2) computed from Hagedorn wavepacket dynamics in the adiabatic harmonic model; a scaling factor of 0.97 was applied to the wavenumbers of the computed spectra (red dashed line); the experimental reference (black solid line) is taken from ref 3.

In Figure 1, the computed and experimental SVL fluorescence spectra are shown with respect to the wavenumber differences ν̃_exc_ – ν̃_em_ between the initial excitation light and the emitted light. An empirical scaling factor was applied to the wavenumber differences of the simulated spectra since the density functional theory (DFT) calculations systematically overestimate the vibrational frequencies of the ground electronic state.^14,86,87^ By optimizing the alignment of peak positions between the simulated and experimental spectra, we set the scaling factor to 0.97, which is close to the vibrational scaling factors reported for the PBE0 functional with similar basis sets.^88^

The simulated spectra from both singly excited levels (first row in Figure 1) agree well with the experiment and are consistent with the results obtained by Tapavicza using a generating function approach (see Figure S2). Both Tapavicza’s approach and our Hagedorn method are exact in harmonic models and are thus equivalent for singly excited levels. The overall structure of the 12^1^ spectrum is reproduced, including the progression of mode 12 (12_*j*_^1^, *j* = 0, 1, 2, 3) and the transitions involving a mode change to another a_g_ mode (e.g., 12_0_^1^6_1_^0^) or combinations with other a_g_ modes (e.g., 12_1_^1^6_1_^0^ and 12_2_^1^6_1_^0^). While the intensity of the 12_0_^1^ peak is overestimated, the computed intensities of certain combination bands (for example, 12_1_^1^7_1_^0^, 12_1_^1^6_1_^0^ and 12_2_^1^6_1_^0^) are significantly lower than experimentally observed. From the 1̅1̅^1^ level, the 1̅1̅_0_^1^ transition is negligible in the experimental spectrum and entirely absent in the computed spectrum, as expected from symmetry considerations. Whereas the harmonic Hagedorn dynamics reproduces the cluster of combination bands between 1500 and 2500 cm^–1^, the peaks of 1̅1̅_0_^1^1̅0̅_1_^0^, 1̅1̅_0_^1^1̅0̅_1_^0^12_1_^0^, and 1̅1̅_0_^1^1̅0̅_1_^0^6_1_^0^ transitions are severely underestimated or missing in the computed spectrum. The harmonic model allows clear peak assignments in the computed spectra. For example, while the 1̅1̅_0_^1^1̅0̅_1_^0^6_1_^0^ and 1̅1̅_1_^1^4_1_^0^ peaks are overlapping in the experiment, the peak present at 1954 cm^–1^ (2015 cm^–1^ before wavenumber scaling) in the computed 1̅1̅^1^ spectrum can be unambiguously assigned to the 1̅1̅_1_^1^4_1_^0^ instead of 1̅1̅_0_^1^1̅0̅_1_^0^6_1_^0^ transition based on the ab initio vibrational frequencies.

In contrast to the alternative method from ref 14, the Hagedorn approach makes it possible to compute SVL spectra from multiply excited levels and using the same trajectory as that already needed for the ground-level emission. The ab initio Hagedorn results for levels 12^2^ and 1̅1̅^2^ (second row in Figure 1) agree reasonably well with the experiments. The computed 12^2^ spectrum correctly captures the experimentally observed decrease (compared to the 12^1^ spectrum) in the intensities of the 12_0_^*j*^, 12_*j*_^*j*^, and 12_*j*_^*j*^6_1_^0^ peaks.

The experimental SVL spectra from 1̅1̅^1^ and 1̅1̅^2^ levels are broadly similar in structure. Symmetry requires that the initial and final total vibrational quantum numbers in the b_1g_ modes maintain the same parity. However, a parity-allowed transition may still have little or no intensity. For example, although allowed by symmetry, the 1̅1̅_0_^2^ transition (a_g_ → a_g_) appears in neither the experimental nor the computed spectrum. Instead, the overall 1̅1̅^2^ spectrum is “shifted” compared to the 1̅1̅^1^ spectrum, with the 1̅1̅_2_^2^ transition serving as a false origin. The computed 1̅1̅^2^ spectrum, like the 1̅1̅^1^ spectrum, fails to describe certain combination peaks (e.g., 1̅1̅_1_^2^1̅0̅_1_^0^ and 1̅1̅_1_^2^1̅0̅_1_^0^12_1_^0^).

As demonstrated in ref 43, the Hagedorn wavepacket dynamics is exact in harmonic potentials and can treat mode distortion (frequency changes) and mode mixing (Duschinsky rotation) exactly. Therefore, the differences between experimental and simulated spectra must be due to the inaccuracies of the global harmonic model from electronic structure calculations, neglect of Herzberg–Teller contributions (particularly for b_1g_ levels), significant anharmonicity of the true potential energy surface, or possibly (but unlikely) experimental error. To better understand the discrepancies, we show below the effects of different global harmonic approximations and the influence of Duschinsky coupling on the computed SVL spectra of anthracene. The importance of anharmonicity is also examined by comparing spectra from vertical, adiabatic, and local harmonic approximations.

Figure 2 shows the effects of displacement, mode distortion, and mode mixing on the computed 12^2^ spectrum. In the first row, we assumed the same harmonic potential energy surface for both excited- and ground-state surfaces (differing only by the adiabatic electronic energy gap). The vibrational eigenfunctions with different vibrational quantum numbers are then orthogonal, and the simple selection rule in this vertically displaced harmonic system results in only a single peak (corresponding to the 12_2_^2^ transition) in the spectrum.

**Figure 2:**
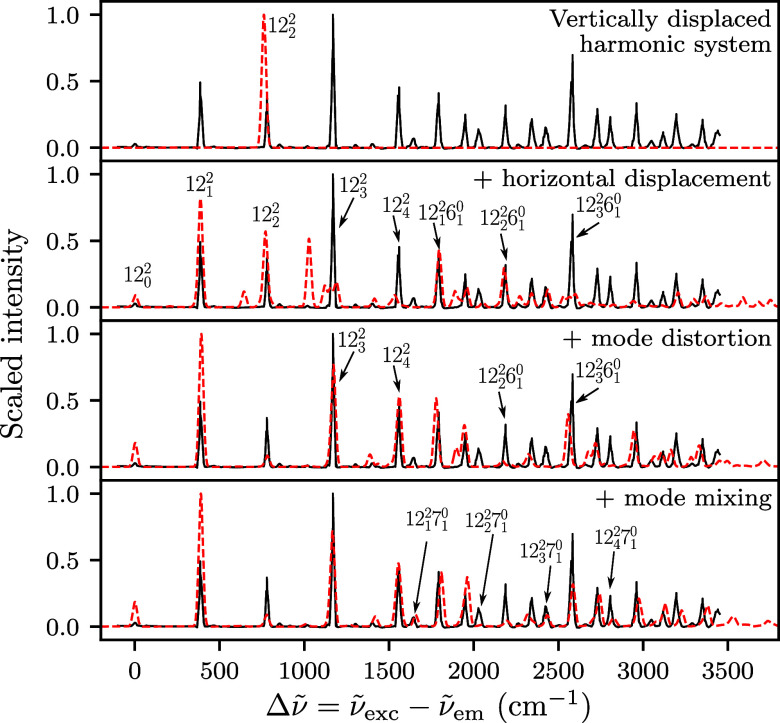
Effects of displacement, mode distortion, and mode mixing on the simulated (red dashed line) 12^2^ SVL fluorescence spectra of anthracene; a scaling factor of 0.97 was applied to the wavenumbers of the computed spectra. All spectra are compared to experiment (ref 3, black solid line).

The displacement of the equilibrium position of the ground-state surface from the excited-state equilibrium produces more interesting, nontrivial spectral features (second row of Figure 2). In the vertically and horizontally displaced harmonic oscillator model, the wavepacket was propagated with a potential centered around the ground-state equilibrium geometry but whose Hessian is identical to the excited-state Hessian (i.e., κ_*g*_ = κ_*e*_ in *V*_*g*_). Several experimental features (e.g., 12_0_^2^, 12_1_^2^, 12_2_^2^, 12_1_^2^6_1_^0^, and 12_2_^2^6_1_^0^) are now reproduced, but some peaks (e.g., 12_3_^2^, 12_4_^2^, and 12_3_^2^6_1_^0^) are underestimated or not resolved in the computed spectrum. The description of the 12_3_^2^, 12_4_^2^, and 12_3_^2^6_1_^0^ peaks is improved in the third row by including mode distortion in the ground state (though the intensity of the 12_2_^2^6_1_^0^ peak becomes worse). Mode distortion, which allows frequency changes between the ground- and excited-state surfaces, was included by defining the ground-state potential using only the diagonal components of the computed ground-state Hessian at the ground-state equilibrium geometry. As the off-diagonal Hessian elements were set to zero, the mode mixing effects were completely neglected in the computed spectrum shown in the third row.

Finally, the fourth row of Figure 2 (the same as the bottom left panel of Figure 1) shows the computed spectrum when the mode mixing is also taken into account. This most comprehensive harmonic description of the potential energy surface (as computed by DFT) improves agreement with experimental data, particularly for transitions with combination levels, for example, the 12_*j*_^2^7_1_^0^ (*j* = 1, 3, 4) peaks, although the intensities are not perfect. The 12_2_^2^7_1_^0^ transition also appears with minimal intensity in the computed spectrum; its extremely low intensity, despite the inclusion of mode mixing, is partly because the 12_2_^2^ transition is underestimated in our harmonic model.

The large observed effects of mode mixing on combination band intensities suggest that the tendency of our ab initio results to underestimate the intensities of certain combination transitions is due to an inaccurate description of Duschinsky coupling between the involved modes. Indeed, the author of ref 14 improved simulated SVL spectra by empirically fitting Duschinsky rotation matrices between several *a*_g_ modes. To further investigate the deficiencies of the 1̅1̅ spectra computed with the ab initio harmonic Hagedorn dynamics, we applied an artificial Duschinsky rotation between modes 1̅1̅ and 1̅0̅ in the computed Hessian matrix (see the Supporting Information for details). As shown in Figure 3, the 1̅1̅^1^ spectrum (top right) evaluated with this modified potential recovers the transitions (1̅1̅_0_^1^1̅0̅_1_^0^, 1̅1̅_0_^1^1̅0̅_1_^0^12_1_^0^, and 1̅1̅_0_^1^1̅0̅_1_^0^6_1_^0^) absent in the original results (top left) and reproduces the experimental intensities of these peaks well. The previously missing peaks (1̅1̅_1_^2^1̅0̅_1_^0^ and 1̅1̅_1_^2^1̅0̅_1_^0^12_1_^0^) in the computed 1̅1̅^2^ spectrum (bottom left) also appear when the artificial coupling is applied (bottom right), but the intensities of the recovered peaks are not as accurate. The results confirm that underestimated mode coupling between vibrational modes is likely the primary cause of the missing peaks in the SVL spectra from 1̅1̅ levels.

**Figure 3:**
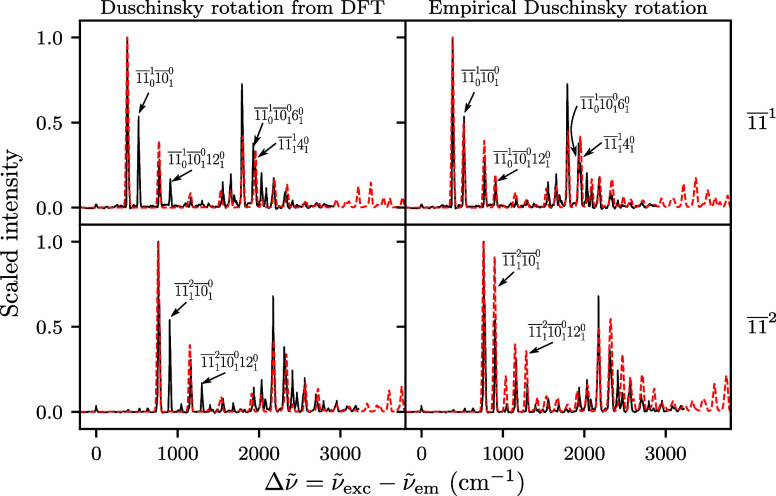
Effects of an artificially enhanced Duschinsky coupling between modes 1̅1̅ and 1̅0̅ on the computed 1̅1̅^1^ and 1̅1̅^2^ SVL fluorescence spectra of anthracene; a scaling factor of 0.97 was applied to the wavenumbers of the computed spectra (red dashed line); the experimental reference (black solid line) is taken from ref 3.

The need for empirical wavenumber scaling in our results (see the Supporting Information for corresponding spectra without wavenumber scaling) indicates that anharmonicity may also be important. To test the adequacy of the global harmonic model, we first computed spectra with the vertical harmonic model where the ground-state surface is expanded around the excited-state equilibrium geometry (Franck–Condon point) instead of the ground-state minimum energy point. If the potential energy surface were completely harmonic, the two different harmonic approximations should give identical results. Figure 4 shows that the 12^2^ SVL spectrum obtained from the vertical harmonic model has notable differences in peak intensities and positions compared to the adiabatic harmonic spectrum (top left). Although the overall structure remains similar, the adiabatic harmonic spectrum (bottom left, Figure 1) provided better agreement with the experiment than the vertical harmonic one (bottom left, Figure 4).

**Figure 4:**
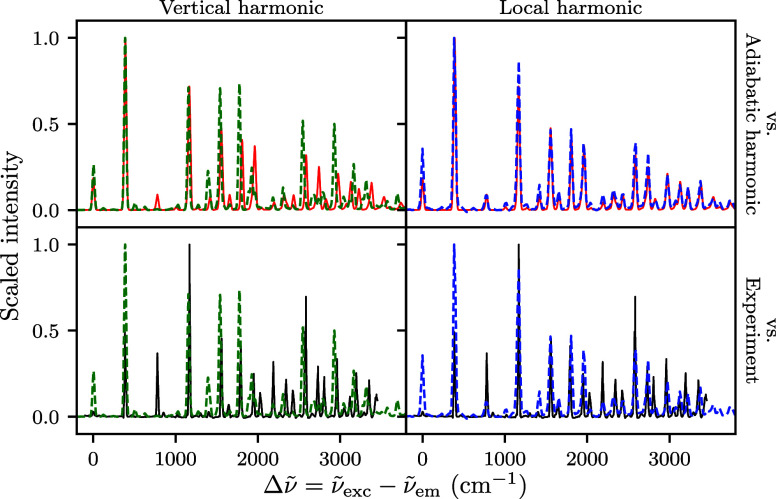
12^2^ SVL fluorescence spectra of anthracene evaluated with the vertical (left, green dashed line) and local harmonic (right, blue dashed line) approaches. The spectra are compared to the adiabatic harmonic (first row, red solid line) and the experimental (ref 3, second row, black solid line) spectra; a scaling factor of 0.97 was applied to all computed spectra.

To confirm the impact of anharmonicity in anthracene, we also performed the much more expensive on-the-fly ab initio local harmonic calculations.^62^ Here, we expand the potential to second order based on DFT evaluations of gradients and Hessians at the current position, rather than using a state-independent global harmonic potential. The center of the wavepacket then follows a fully anharmonic classical trajectory, while the evolution of the width (matrices *Q*_*t*_ and *P*_*t*_) is determined by the local Hessian at each time step. The local harmonic approximation produced very similar results to the global adiabatic harmonic approach (top right, Figure 4). Figure 5 compares the position evolution in mode 12 for the three approximations. The adiabatic and local harmonic trajectories are nearly identical with small differences in the amplitude of motion, whereas the vertical harmonic model gives a significantly different trajectory in the amplitude and frequency of oscillation. This suggests that the (adiabatic) harmonic model suffices for the evaluation of SVL spectra of anthracene.

**Figure 5:**
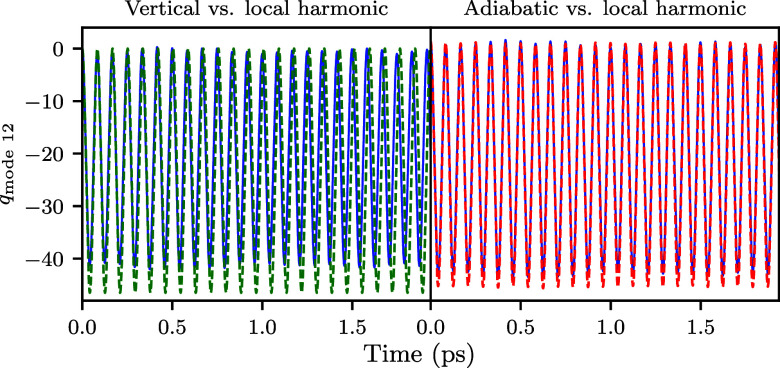
Evolution of the position along mode 12 (*q*_mode 12_) of the center of the wavepacket in the on-the-fly local harmonic dynamics (blue solid line), compared to the vertical (left panel, green dashed line) and adiabatic (right panel, red dashed line) harmonic dynamics.

However, empirical wavenumber scaling is still required for the local harmonic results to achieve good agreement with experimental wavenumbers (bottom right, Figure 4). Although the use of scaling factors are typically attributed to anharmonicity, our results suggest they may be primarily compensating for systematic bias (e.g., due to electronic correlation) in the chosen DFT method. While outside the scope of the present work focused on the dynamics method, further studies using anharmonic frequency corrections (e.g., with perturbation theory^89,90^ ) could help separate electronic structure errors from genuine anharmonic contributions. The sensitivity of the computed spectra to the different harmonic models in Figures 2–4 reflects how vibronic spectra may reveal detailed information about molecular potential energy surfaces in experiments.

We also note that the additional cost of computing overlaps for different initial states is negligible compared to propagation in the local harmonic case. As a rough comparison, in the global harmonic model, propagating the parameters of the Gaussian for 100,000 steps took approximately 12 CPU minutes (single-core) in total, while computing 25,000 overlaps for a doubly excited level required around 72 CPU minutes on the same computer. Whereas constructing the global harmonic potential for anthracene required only a single electronic structure evaluation (approximately 90 CPU minutes), on-the-fly local harmonic dynamics required performing such a calculation at *each* time step.

Since the overlap expressions for two Hagedorn functions are general for any non-negative multi-index *K*, our method can use the same trajectory to treat not only multiple excitations in a single mode, but also initial states where several vibrational modes are simultaneously excited to any vibrational level (although the cost of computing overlaps increases as more modes are excited to higher levels). Here, we have restricted ourselves to the experimentally accessible vibronic levels (12^1^6^1^, 12^1^5̅^1^, and 12^1^5^1^), which were reported respectively at 1380 + 385, 1409 + 385, and 1420 + 385 cm^–1^ in the experimental fluorescence excitation spectrum of anthracene, albeit with very weak intensities (2–3% of the signal at origin).^3^ Despite the current lack of experimental data on the SVL fluorescence from these levels for a direct comparison, the Hagedorn approach can serve as a tool for predicting complex vibronic spectra.

Figure 6 shows the SVL emission spectra from the levels 12^1^6^1^, 12^1^5̅^1^, and 12^1^5^1^ predicted with the adiabatic harmonic model. These spectra are broadly similar to the simulated 12^1^ spectrum, but the excitation in an additional mode leads to different intensity patterns. Compared to the 12^1^6^1^ spectrum, the predicted spectrum from the 12^1^5^1^ level shows weaker intensities in the first three peaks corresponding to a progression in mode 12. In contrast to 12^1^6^1^ and 12^1^5^1^ spectra, in the 12^1^5̅^1^ spectrum the three peaks of the mode 12 progression under 1000 cm^–1^ (i.e., 12_*j*_^1^5̅_0_^1^ with *j* = 0, 1, 2) disappear, because transitions to a_g_ levels (e.g., the ground level and 12_*j*_ levels) in the ground state are forbidden for initial levels of b_1g_ symmetry. The progression in mode 12 is only allowed when coupled with the symmetry-allowed 5̅_1_^1^ transition (e.g., 12_0_^1^5̅_1_^1^, 12_1_^1^5̅_1_^1^, and 12_2_^1^5̅_1_^1^). Otherwise, the features above 2500 cm^–1^ (mainly combination bands with other a_g_ modes, analogous to the >1000 cm^–1^ peaks in the 12^1^ spectrum) are similar in the SVL spectra from 12^1^5̅^1^ and 12^1^5^1^ levels.

**Figure 6:**
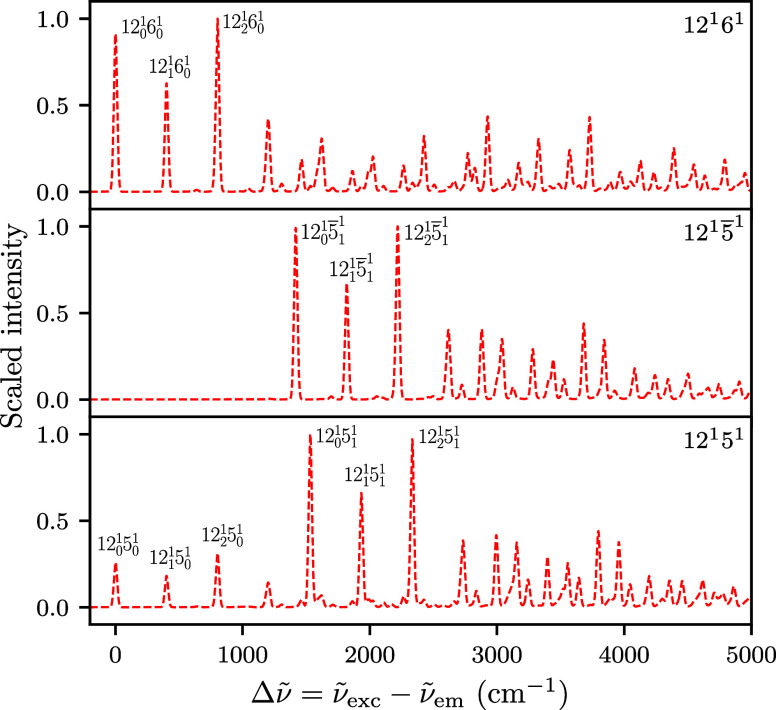
SVL fluorescence spectra of anthracene from initial vibrational levels 12^1^6^1^, 12^1^5̅^1^, and 12^1^5^1^ predicted with Hagedorn wavepacket dynamics in the adiabatic harmonic model; a scaling factor of 0.97 was applied to the wavenumbers.

## 5 Conclusions

To conclude, we have combined Hagedorn wavepacket dynamics with DFT evaluation of the electronic structure in order to simulate SVL spectroscopy in a realistic molecular system. From a single semiclassical trajectory, we were able to efficiently compute SVL spectra of anthracene beyond emission from singly excited vibrational levels and consistent with symmetry considerations and in good agreement with experiments.

Because the vibrational Hagedorn dynamics in the global harmonic model is exact, it is easier to identify and isolate errors. Our analysis of the results with different harmonic approximations demonstrates the importance of including both mode distortion and Duschinsky rotation in evaluating molecular vibronic spectra, which serve as a sensitive probe for molecular structure and dynamics. For anthracene, our results suggest that the electronic structure methods we used underestimated mode mixing, which affected the accuracy of combination band intensities.

The Hagedorn approach can be readily extended to capture mild anharmonic effects through the local harmonic approximation, which requires expensive on-the-fly electronic structure evaluations along the trajectory (though the same propagation can again be used to evaluate spectra from all initial excitations).^62^ Computing vibronic spectra within different global harmonic models can help selecting appropriate ab initio methods and identifying when anharmonicity becomes significant before more sophisticated quantum dynamics methods are applied.

Even within the global harmonic framework, an extension of our method could be useful for other experimental techniques involving vibrationally excited states, such as vibrationally promoted electronic resonance (VIPER) experiments,^91–93^ time-resolved photoelectron spectroscopy,^94^ and fluorescence-encoded infrared (FEIR) spectroscopy.^95^ More generally, Hagedorn functions form a complete orthonormal basis and can exactly expand arbitrary polynomials times a Gaussian.^46,53^ This could enable Hagedorn wavepackets to treat more complex initial states, for example, in non-Condon spectroscopy.^30,37,92,96^ Finally, to account for nonadiabatic effects, one could extend the approach by variationally propagating multiple Hagedorn bases on coupled electronic surfaces.^56,97–99^

## Supplementary Material



## Data Availability

In addition to the data provided in the article and the Supporting Information, the electronic structure results used to construct the initial state and the global harmonic models, along with the computed spectra and autocorrelation functions, are available on Zenodo (DOI: 10.5281/zenodo.15693437).

## Notes

Expressions to compute the overlaps between Hagedorn functions, optimized *S*_0_ and *S*_1_ geometries of anthracene with computed and experimental frequencies of selected vibrational modes, additional computational details of the empirical Duschinsky coupling and of the local harmonic calculation, computed and experimental 0^0^ emission spectra, and computed spectra in Figures 1 and 4 without wavenumber scaling (PDF)
