# Supplementary material for: Simulating Molecular Single Vibronic Level Fluorescence Spectra with Ab Initio Hagedorn Wavepacket Dynamics
Source: J Chem Theory Comput. 2025 Sep 15;21(19):9726–35. doi: 10.1021/acs.jctc.5c01097 (PMC12529895; doi:10.1021/acs.jctc.5c01097)
Supplement: Supplementary file 1 [file ct5c01097_si_001.pdf]

Supporting Information for Simulating Molecular Single Vibronic Level Fluorescence Spectra with ab initio Hagedorn Wavepacket Dynamics

Zhan Tong Zhang and Jiří J. L. Vaníček*

Laboratory of Theoretical Physical Chemistry, Institut des Sciences et Ingénierie Chimiques, Ecole Polytechnique Fédérale de Lausanne (EPFL), CH-1015 Lausanne, Switzerland

E-mail: jiri.vanicek@epfl.ch

1 Expressions to compute overlaps between two Hagedorn functions associated with different Gaussians

The expressions used to compute the overlap $M_{JK'} = \langle J|K'\rangle = \langle \varphi_J|\varphi_{K'}\rangle$ between two Hagedorn functions $\varphi_J := \varphi_J[\Lambda]$ and $\varphi_{K'} := \varphi_K[\Lambda']$, associated with two Gaussians characterized by two different sets of parameters $\Lambda = (q, p, Q, P, S)$ and $\Lambda' = (q', p', Q', P', S')$, were derived in ref 61 (Proposition 10) and are reproduced below. We use the bar (e.g., in $\overline{P}$) to denote the complex conjugate and the dagger (e.g., in $U^\dagger$) to denote the conjugate transpose.

A code to compute the overlaps is provided on Zenodo (DOI: 10.5281/zenodo.14332258) and in the supplementary material of ref 43.

1.1 Auxiliary matrices and vectors

First, we define matrices

$$U(\Lambda, \Lambda') := \frac{i}{2}\left(Q'^T \cdot \overline{P} - P'^T \cdot \overline{Q}\right), \tag{1}$$

$$V(\Lambda, \Lambda') := \frac{i}{2}\left(Q'^T \cdot P - P'^T \cdot Q\right), \tag{2}$$

and vectors

$$v\left(\Lambda,\Lambda'\right) := \frac{i}{\sqrt{2\hbar}}\left[Q'^{T}\cdot(p_t - p'_t) - P'^{T}\cdot(q_t - q'_t)\right], \tag{3}$$

$$v'\left(\Lambda,\Lambda'\right) := \frac{i}{\sqrt{2\hbar}}\left[Q^{T}\cdot(p'_t - p_t) - P^{T}\cdot(q'_t - q_t)\right], \tag{4}$$

which relate ladder operators associated with two different Gaussians via the Bogoliubov transformation (see sec. 3.2 in ref 61).

We then define the auxiliary matrices

$$W := \left(U^{\dagger}\cdot U\right)^{-1}, \tag{5}$$

$$W' := \left(\overline{U}\cdot U^{T}\right)^{-1}, \tag{6}$$

and vectors

$$w := -V^{T}\cdot\overline{v} + v', \tag{7}$$

$$w' := \overline{V}\cdot v' + \overline{v}. \tag{8}$$

1.2 Recurrence relations to compute overlaps between two Hagedorn functions

The overlaps between two Hagedorn functions may be computed from the recurrence relations (Proposition 10 in ref 61)

$$\sqrt{J_j+1}M_{J+\langle j\rangle,K'} = \sum_{k=1}^{D}\left(F_{jk}\sqrt{K'_k}M_{J,K'-\langle k\rangle} - G_{jk}\sqrt{J_k}M_{J-\langle k\rangle,K'}\right) + u_j M_{JK'}, \tag{9}$$

$$\sqrt{K'_k+1}M_{J,K'+\langle k\rangle} = \sum_{j=1}^{D}\left(G'_{kj}\sqrt{K'_j}M_{J,K'-\langle j\rangle} + F'_{kj}\sqrt{J_j}M_{J-\langle j\rangle,K'}\right) + u'_k M_{JK'}, \tag{10}$$

which eventually reduce to the overlap $M_{00'}$ (see eq 80 in ref 61) between the two Gaussian centers $\varphi_0[\Lambda]$ and $\varphi_0[\Lambda']$. Here, the expressions are simplified using the auxiliary matrices

$$F := W\cdot U^{\dagger} \text{ and } G := W\cdot V^{T}\cdot\overline{U}, \tag{11}$$

$$F' := W'\cdot\overline{U} \text{ and } G' := W'\cdot\overline{V}\cdot U^{\dagger}, \tag{12}$$

and vectors

$$u := W\cdot w, \tag{13}$$

$$u' := W'\cdot w'. \tag{14}$$

2 Optimized structures

The optimized structures are provided in Cartesian coordinates and in atomic units (Bohr).

2.1 Ground electronic state S_0

C	6.85759734	0.93861618	0.18661114
C	6.75317532	-1.73927774	0.22839486
C	4.48701231	-2.95454121	0.24041903
C	2.18052437	-1.57623417	0.21156067
C	4.69288950	2.32632695	0.15801737
C	2.28611471	1.13145888	0.16931033
C	-0.16472798	-2.76711373	0.22298903
C	-2.41019684	-1.39756084	0.19446137
C	0.04065360	2.50100820	0.14077883
C	-2.30461136	1.31012665	0.15220881
C	-4.81697128	-2.59242925	0.20574956
C	-6.98167438	-1.20471842	0.17720160
C	-6.87726018	1.47317070	0.13545140
C	-4.61109328	2.68843865	0.12337971
H	8.67959788	1.87504544	0.17756881
H	8.49674521	-2.81452270	0.25072195
H	4.40284123	-5.00304096	0.27236425
H	4.76842585	4.37516499	0.12604093
H	-0.24464769	-4.81764931	0.25501229
H	0.12058213	4.55153639	0.10877692
H	-4.89250968	-4.64126986	0.23771316
H	-8.80367254	-2.14115635	0.18623662
H	-8.62082483	2.54841398	0.11316999
H	-4.52693174	4.73693790	0.09143082

2.2 Excited electronic state S_1

C	6.93429995	0.90129360	0.18766897
C	6.83292916	-1.70649147	0.22828749
C	4.48716391	-2.93861954	0.24027276
C	2.22056696	-1.57650330	0.21174682
C	4.69123361	2.31148031	0.15843761
C	2.32575534	1.12913475	0.16951553
C	-0.16392966	-2.75418575	0.22283665
C	-2.44983475	-1.39524217	0.19427032
C	0.03984978	2.48807841	0.14083596
C	-2.34464687	1.31039760	0.15194638
C	-4.81531370	-2.57758329	0.20551568
C	-7.05838102	-1.16739708	0.17630561

```
C   -6.95701147   1.44038446   0.13550575
C   -4.61124374   2.67251203   0.12339667
H    8.74000200   1.86647402   0.17822684
H    8.55826550  -2.80873254   0.25097962
H    4.41235101  -4.98723381   0.27212626
H    4.77565479   4.35972098   0.12653071
H   -0.24366543  -4.80548476   0.25497786
H    0.11958926   4.53937715   0.10864797
H   -4.89973558  -4.62582283   0.23746899
H   -8.86408258  -2.13257685   0.18592208
H   -8.68234629   2.54262590   0.11274910
H   -4.53643253   4.72112455   0.09139783
```

3 Vibrational frequencies

3.1 Ground electronic state S_0

Table S1: Computed (DFT) and experimental (Exp., ref 3 unless otherwise noted) S_0 vibrational frequencies of modes 4, 6, 7, 12, $\overline{10}$, and $\overline{11}$. [a] Value from Räsänen, J; Stenman, F.; Penttinen, E., *Spectrochim. Acta A Mol. Spectrosc.* **1973**, *29*, 395

**Mode label**	**Symmetry**	**DFT (cm^{-1})**	**Exp. (cm^{-1})**
4	a_g	1623.0	1566
6	a_g	1462.5	1408
7	a_g	1306.4	1263
12	a_g	400.9	390
$\overline{10}$	b_{1g}	531.4	524
$\overline{11}$	b_{1g}	391.8	391[a]

3.2 Excited electronic state S_1

Table S2: Computed (DFT) and experimental (Exp., ref 3) S_1 vibrational frequencies of modes 5, 12, and $\overline{11}$.

**Mode label**	**Symmetry**	**DFT (cm^{-1})**	**Exp. (cm^{-1})**
5	a_g	1546.4	1420
12	a_g	382.6	385
$\overline{11}$	b_{1g}	396.1	232

4 Computational details

4.1 Empirical Duschinsky rotation (fig 4)

A rotation matrix $R(\theta) = \begin{pmatrix} \cos\theta & -\sin\theta \\ \sin\theta & \cos\theta \end{pmatrix}$ was applied to the submatrix $\kappa_{g,\mathrm{DFT}}^{\overline{11},\overline{10}}$ of modes $\overline{11}$ and $\overline{10}$ of the ground-state Hessian matrix $\kappa_{g,\mathrm{DFT}}$ (obtained from the ab initio calculation) in the excited-state normal-mode coordinates to obtain the modified Hessian $\kappa_{g,\mathrm{empirical}}$ with $\kappa_{g,\mathrm{empirical}}^{\overline{11},\overline{10}} = R(\theta)^T \cdot \kappa_{g,\mathrm{DFT}}^{\overline{11},\overline{10}} \cdot R(\theta)$. This artificially enhanced Duschinsky coupling produced the "empirically" rotated system with the rotation angle $\theta = 31.5°$, whose SVL spectra are shown in the right column of fig 3 in the main text.

4.2 On-the-fly local harmonic approximation

The parameters of the Gaussian were propagated with a second-order TVT geometric integrator for a total time of 8×10^4 au (∼1.9 ps) with a time step of 8 au. At each time step, the position in normal-mode coordinates was converted to Cartesian coordinates for ab initio calculations. The potential energy, its gradient, and its Hessian were evaluated at each point along the classical trajectory with Gaussian 16 at the same level of theory (PBE0/def2-TZVP) as the global harmonic models; the values were then converted to the excited-state normal-mode coordinates for propagation of the Gaussian.

The autocorrelation functions of the Hagedorn wavepackets (associated with the local harmonic trajectory of the Gaussian) were computed every four steps using the algebraic algorithm described in ref 60. A Gaussian damping function with a half-width at half-maximum of 20000 au was applied to the autocorrelation functions before performing the Fourier transform.

5 0^0 ground-level emission spectrum

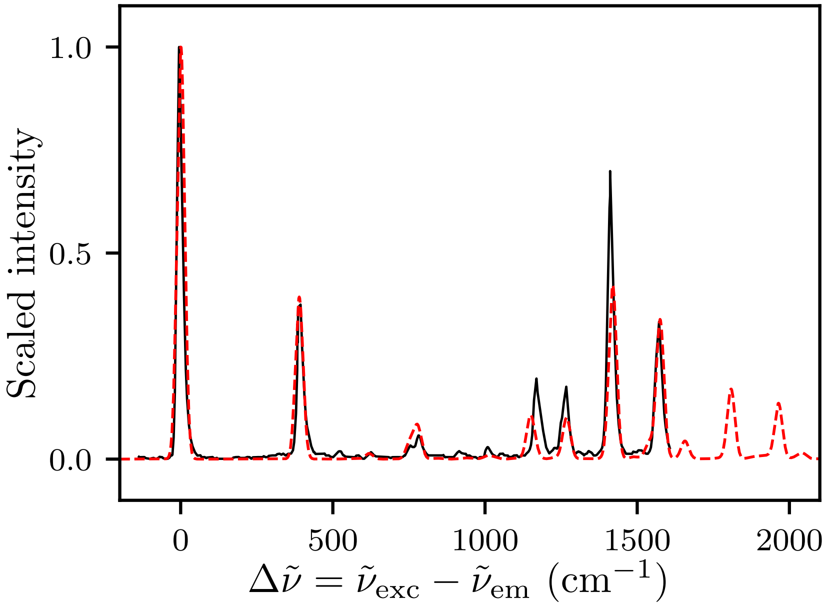

Figure S1: Ground-level (0^0) fluorescence spectra of anthracene computed from Gaussian wavepacket dynamics in the adiabatic harmonic model (red dashed line); the computed wavenumbers were scaled by a factor of 0.97; the experimental reference (black solid line) is taken from ref 3.

6 Comparison with results from ref 14

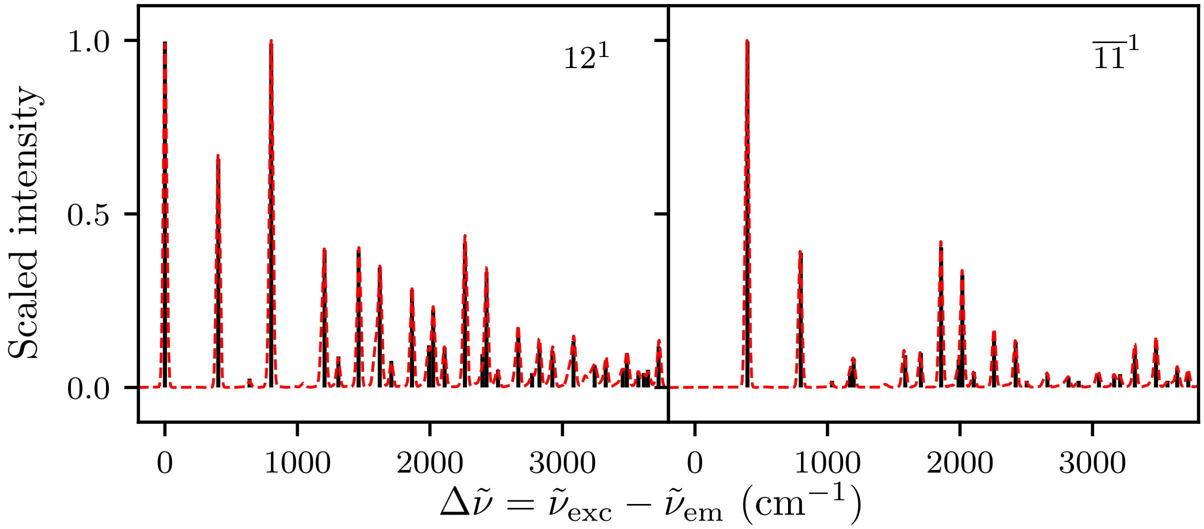

Figure S2: SVL fluorescence spectra of anthracene from levels 12^1 and $\overline{11}^1$ computed from Hagedorn wavepacket dynamics (red dashed line) compared to the spectra computed using the generating function approach by Tapavicza (black sticks, ref 14) in the PBE0 adiabatic harmonic model.

7 SVL emission spectra without wavenumber scaling

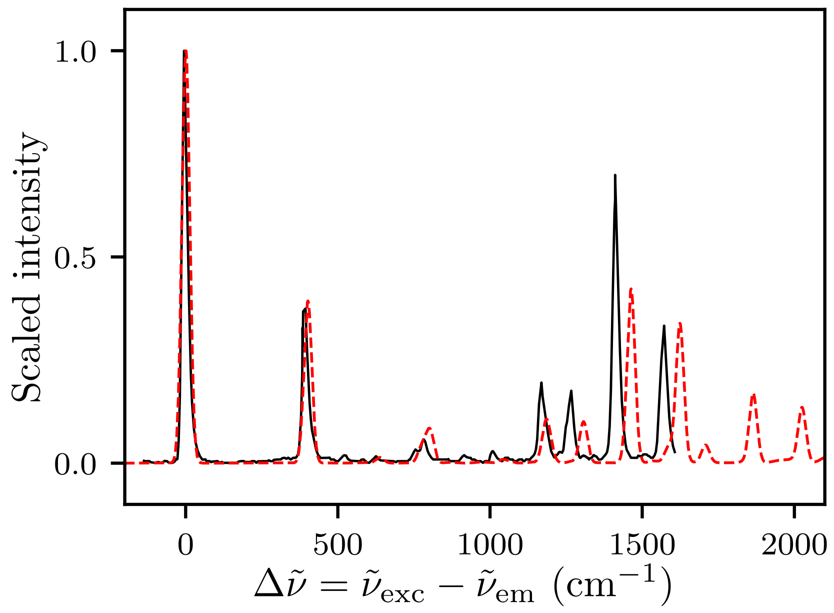

Figure S3: Ground-level (0^0) fluorescence spectra of anthracene computed from Gaussian wavepacket dynamics in the adiabatic harmonic model, without wavenumber scaling (otherwise the same as fig S1, red dashed line); the experimental reference (black solid line) is taken from ref 3.

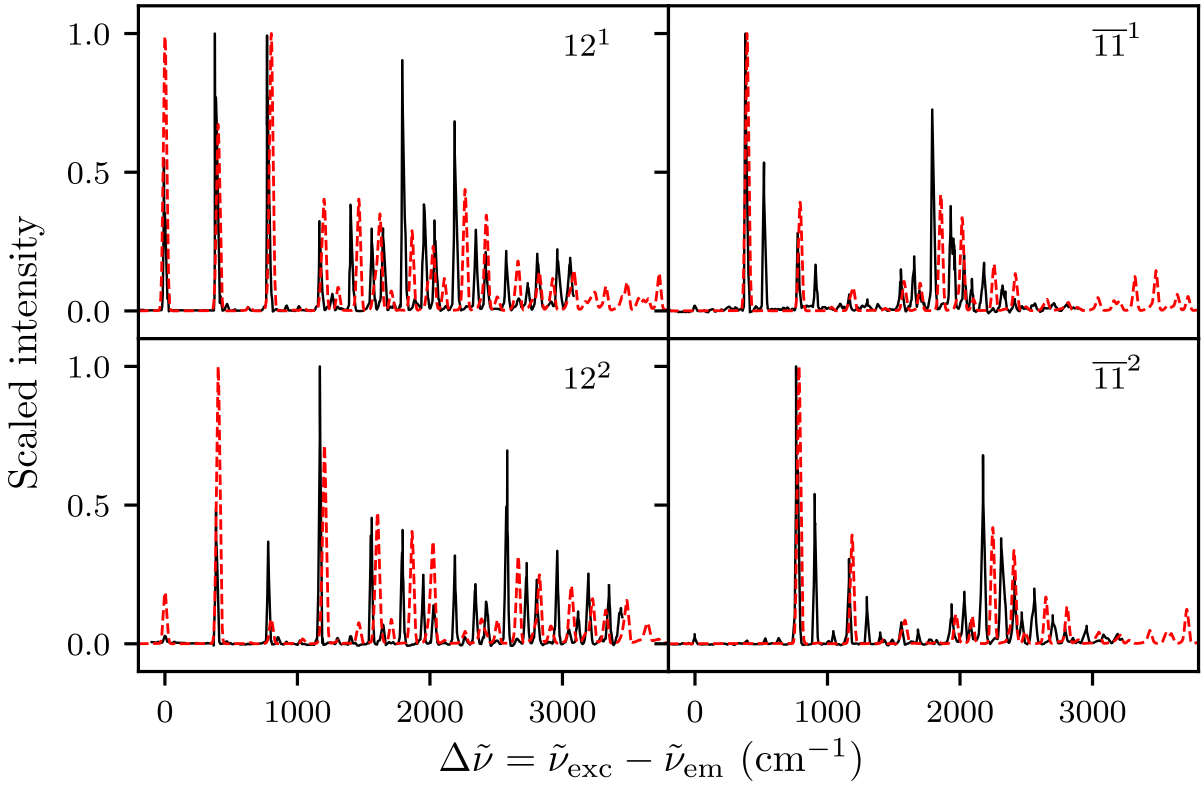

Figure S4: SVL fluorescence spectra of anthracene from initial vibrational levels 12^j and $\overline{11}^j$ ($j = 1, 2$) computed from Hagedorn wavepacket dynamics in the adiabatic harmonic model, without wavenumber scaling (otherwise the same as fig 1 of the main text, red dashed line); the experimental reference (black solid line) is taken from ref 3.

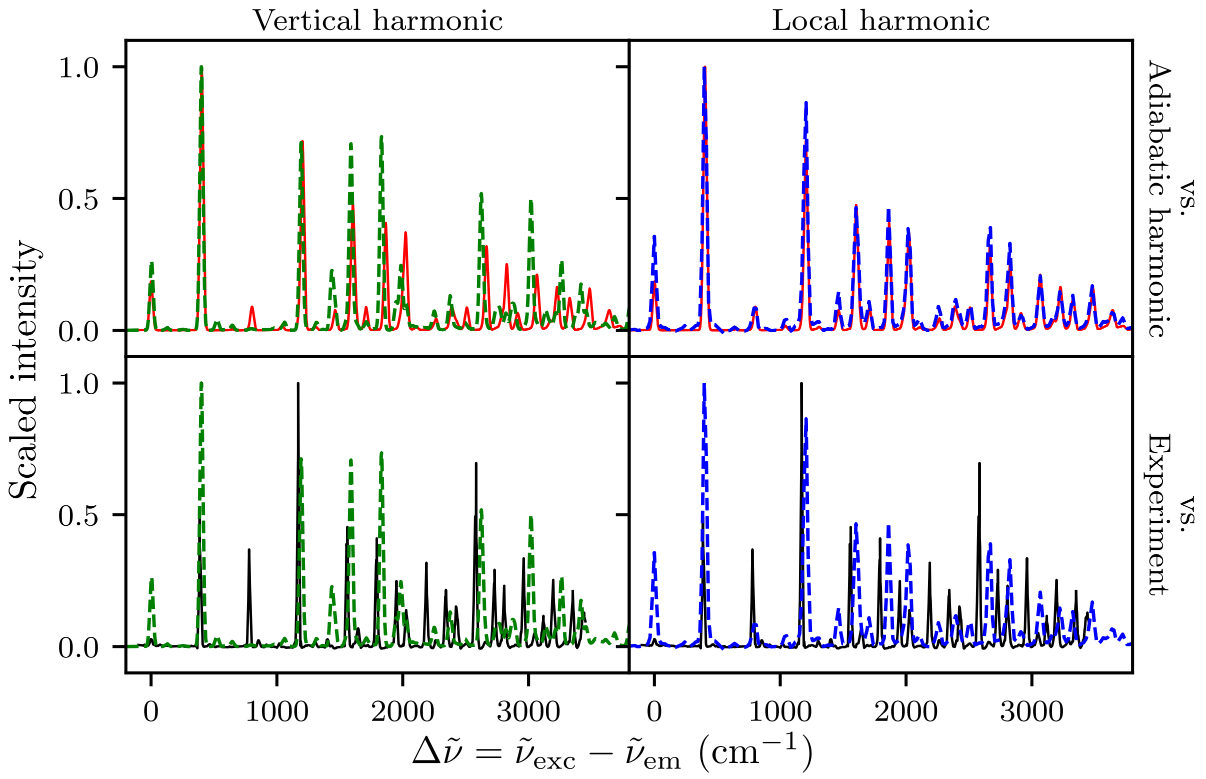

Figure S5: 12^2 SVL fluorescence spectra of anthracene evaluated with the vertical (left, green dashed line) and local harmonic (right, blue dashed line) approaches, without wavenumber scaling (otherwise the same as fig 4 of the main text). The spectra are compared to the adiabatic harmonic (first row, red solid line) and the experimental (ref 3, second row, black solid line) spectra.
